# Severe temozolomide-induced thrombocytopenia is linked to increased healthcare utilization in glioblastoma and disproportionally impacts female patients

**DOI:** 10.1093/nop/npaf013

**Published:** 2025-01-22

**Authors:** Leon van Hout, Alessia D Borgo, Nienke Grun, Maaike Schuur, Martijn P G Broen, Bart A Westerman, Imke Bartelink, William Peter Vandertop, Birgit I Lissenberg – Witte, Mathilde C M Kouwenhoven

**Affiliations:** Cancer Center Amsterdam, Treatment and Quality of Life, Amsterdam, The Netherlands; Amsterdam UMC Location Vrije Universiteit Amsterdam, Neurology, Amsterdam, The Netherlands; Amsterdam UMC Location Vrije Universiteit Amsterdam, Neurosurgery, Amsterdam, The Netherlands; Cancer Center Amsterdam, Treatment and Quality of Life, Amsterdam, The Netherlands; Amsterdam UMC Location Vrije Universiteit Amsterdam, Neurology, Amsterdam, The Netherlands; Cancer Center Amsterdam, Treatment and Quality of Life, Amsterdam, The Netherlands; Amsterdam UMC Location Vrije Universiteit Amsterdam, Neurology, Amsterdam, The Netherlands; Stichting Epilepsie Instellingen Nederland (SEIN), Neurology, Heemstede, The Netherlands; Cancer Center Amsterdam, Treatment and Quality of Life, Amsterdam, The Netherlands; Amsterdam UMC Location Vrije Universiteit Amsterdam, Neurology, Amsterdam, The Netherlands; Department of Neurology, GROW School for Oncology and Reproduction, Maastricht University Medical Center, Maastricht, The Netherlands; Cancer Center Amsterdam, Treatment and Quality of Life, Amsterdam, The Netherlands; Amsterdam UMC Location Vrije Universiteit Amsterdam, Neurosurgery, Amsterdam, The Netherlands; Cancer Center Amsterdam; Imaging and Biomarkers, Amsterdam, The Netherlands; Department of Clinical Pharmacy & Pharmacology, Amsterdam, The Netherlands; Cancer Center Amsterdam, Treatment and Quality of Life, Amsterdam, The Netherlands; Cancer Center Amsterdam, Treatment and Quality of Life, Amsterdam, The Netherlands; Amsterdam UMC Location Vrije Universiteit Amsterdam, Neurosurgery, Amsterdam, The Netherlands; Amsterdam UMC Location Vrije Universiteit Amsterdam, Epidemiology and Data Science, Amsterdam, The Netherlands; Cancer Center Amsterdam, Treatment and Quality of Life, Amsterdam, The Netherlands; Amsterdam UMC Location Vrije Universiteit Amsterdam, Neurology, Amsterdam, The Netherlands

**Keywords:** glioblastoma, healthcare utilization, temozolomide, thrombocytopenia

## Abstract

**Background:**

Thrombocytopenia is a major temozolomide-induced adverse event during the standard treatment of glioblastoma. Consequently, platelet transfusions and treatment modifications may impact quality of life and long-term treatment outcomes. Understanding the impact of thrombocytopenia on healthcare utilization is crucial to mitigate the need for healthcare resources in glioblastoma patients. Here, we assess the influence of thrombocytopenia-related healthcare among patients diagnosed with glioblastoma.

**Methods:**

We retrospectively collected patient information treated at the Brain Tumor Center Amsterdam between 2008 and 2021. The occurrence of thrombocytopenia, patient demographics, treatment details, and healthcare utilization data were gathered from patients who received standard glioblastoma treatment. Associations between temporal severity of thrombocytopenia as categorized by the Common Terminology Criteria for Adverse Events, patient characteristics, and healthcare utilization were analyzed using Generalized Linear Mixed Models.

**Results:**

We included 206 patients with a median age of 58 years, 35.9% were female and we found that thrombocytopenia (any grade) occurred in 61.1% of patients. The occurrence of thrombocytopenia during CRT was associated with increased healthcare utilization and was largest in females who developed grade 4 thrombocytopenia compared to those who did not develop thrombocytopenia (OR = 5.9, *P* < .001 in females vs OR = 4.4, *P* < .001 in males). Grade 4 thrombocytopenia was also associated with heightened healthcare utilization during the adjuvant phase (OR = 7.6, *P* < .001), and was comparable between sexes.

**Conclusions:**

Severe thrombocytopenia during glioblastoma treatment is linked to increased healthcare utilization, disproportionally impacting females. These data suggest that prevention and early management of thrombocytopenia can reduce healthcare utilization in patients with glioblastoma.

**Key Points**

- Thrombocytopenia occurs in over half of glioblastoma patients receiving treatment.
- Thrombocytopenia results in increased healthcare utilization.
- Females are mainly affected in healthcare use when thrombocytopenia occurs.

**Importance of the Study**

Chemotherapy-induced thrombocytopenia is common in glioblastoma patients, yet its effect on healthcare utilization remains unstudied. This retrospective cohort study from the Brain Tumor Center Amsterdam examines the association between thrombocytopenia severity and healthcare utilization, focusing on sex-specific disparities. Through detailed analysis of healthcare resource use, patient demographics, and the timing of thrombocytopenia, we explored this association. We found that severe thrombocytopenia increases overall healthcare utilization, leading to higher rates of emergency presentations, longer hospital admissions, and more frequent thrombocyte transfusions. This association disproportionately affects female patients who develop grade 4 thrombocytopenia during chemo-radiotherapy. These findings highlight the need for preemptive strategies and early intervention for thrombocytopenia, particularly in female patients, to reduce healthcare resource strain. Our study enhances the understanding of glioblastoma management and suggests more targeted, efficient, and equitable healthcare approaches.

In newly diagnosed patients with glioblastoma, the standard of care consists of maximal safe surgical resection, followed by radiotherapy with concomitant temozolomide chemotherapy, concluded by adjuvant temozolomide monotherapy^1,2^ Conventional healthcare utilization during the chemo-radiotherapy phase (CRT) consists of one daily radiotherapy session for 30 days, along with weekly checkups and lab exams in the outpatient clinic. Outpatient clinic visits become less frequent and occur once a month during the 6 adjuvant courses of temozolomide.

Unplanned healthcare utilization may arise when patients develop severe tumor symptoms when tumor progression occurs and in the event of treatment-induced adverse events, such as fever, neutropenia, and thrombocytopenia.^3,4^ Studies on these adverse events are particularly relevant for maintaining quality of life and minimizing hospital admissions.^5^ However, the factors, and their impact, contributing to increased healthcare utilization in glioblastoma patients, are scarcely described.

Chemotherapy-induced thrombocytopenia represents a frequent adverse event, affecting 23%–64% of patients undergoing standard treatment for glioblastoma. Of these, 11%–46% are affected during CRT and 10%–64% in the adjuvant phase.^6–8^ The Common Terminology Criteria for Adverse Events criteria (CTCAE, v 5.0) defines thrombocytopenia as a blood platelet count below 150 000/µL, however, the risk of bleeding drastically increases at counts lower than 50 000/µL (grade 3) and are largest beneath 25 000/µL (grade 4).^9,10^ Of glioblastoma patients, 7%–26% experience a severe grade of thrombocytopenia (CTCAE grade 3 or higher), with an up to 4-fold higher incidence in females.^6,11–15^ This sex-based discrepancy in thrombocytopenia occurrence is hypothesized to be the result of a difference in temozolomide clearance between male and female patients.^16,17^ The potential consequences of this adverse event are severe. In different solid tumors treated with alkylating agents, severe thrombocytopenia (grade 3–4) has been linked with increased healthcare costs, the need for platelet transfusions, and hospitalizations.^10,18,19^ Additionally, thrombocytopenia is linked with adjustments to temozolomide dosages or even discontinuation of chemotherapy.^7^ However, the impact of thrombocytopenia on healthcare utilization in patients with glioblastoma has not yet been studied.

We tested the hypothesis that the onset of thrombocytopenia is associated with increased treatment intensity in patients with glioblastoma. We retrospectively compared the effect of thrombocytopenia on the number of hospital interactions (ie, planned, and unplanned encounters) and identified potential risk factors for increased healthcare utilization. Furthermore, we assessed whether the female sex modifies the association between thrombocytopenia and healthcare utilization.

## Methods

### Study Population

Of 1171 patients with a newly identified histologically confirmed glioblastoma, 474 (40.5%) received maximal safe resection of the tumor, 6 weeks of concomitant chemo-radiotherapy and finally 6 cycles of adjuvant chemotherapy between 2008 and 2021 at the Brain Tumor Center Amsterdam. We excluded patients if planned treatment schedules were unclear (commonly seen when patients were treated in multiple hospitals), if other unrelated comorbidities influenced the number of planned hospital interactions during treatment, if patients participated in an experimental treatment, or if most thrombocyte values were missing during treatment. We extracted the following demographic variables for each patient: sex, age, body surface area, preoperative Karnofsky Performance Scale, tumor location, and extent and type of resection. Additionally, concomitant use of proton pump inhibitors, seizure-modifying treatment, and/or corticosteroids were reported as these have previously been linked to the development of thrombocytopenia.^20,21^ Occurrences of vascular adverse events were documented throughout the inclusion period if they developed. Additionally, any periods falling after March 27, 2020, were recorded as pandemic-related interactions, as the COVID-19 pandemic has significantly influenced healthcare utilization globally.^22,23^ The Medical Review Ethics Committee of Amsterdam UMC approved this study (VUMC2020.075).

### Thrombocytopenia and Toxicity Registration

Longitudinal circulating blood cell counts, including thrombocytes, red blood cells, leukocytes, lymphocytes, and neutrophils, were recorded throughout treatment. Myelotoxicity severity was classified based on the Common Terminology Criteria for Adverse Events (CTCAE v. 5.0). Nadir cell count values and the highest myelotoxicity grades were documented for all patients at the following time points: prior to CRT, weekly during the CRT phase, before the adjuvant phase, monthly during the adjuvant phase, and 1-month post-treatment. Additionally, nadir cell counts and corresponding CTCAE grades for each treatment phase were registered.

### Hospital Interactions

Healthcare utilization was quantified by registration of all glioblastoma treatment-related hospital interactions: physical consults and telephone calls with medical specialists (neurosurgeons, neurologists, oncologists, and radiotherapists) and nurse practitioners, the number of and reasons for ER visits and hospital admissions, types of imaging techniques and their occurrences, and the number of the blood draw and thrombocyte transfusions. We distinguished between planned and unplanned hospital interactions by comparing each visit against the patient’s planned treatment schedule. We registered the sum of all hospital interactions that occurred between the resection of the tumor and the start of the CRT phase, weekly during the CRT phase, before the start of the adjuvant phase, and monthly during the adjuvant phase. The primary endpoint for this study was total unplanned hospital interactions during the CRT and adjuvant treatment phases. Secondary endpoints included the number of days spent admitted to the hospital and the number of ER admissions, given that these hospital interactions are among the most time-consuming for patients.

### Statistical Analysis

To analyze demographic differences between patients based on thrombocytopenia grades and sex, we selected appropriate statistical tests based on the data characteristics. Chi-square tests, Fisher’s exact tests, independent sample *t*-tests, and Mann–Whitney *U* tests were used accordingly. Q–Q plots and frequency histograms were visually inspected to determine continuous variable distributions, guiding our choice of tests. Due to the weekly thrombocyte measurements during CRT and monthly measurements during the adjuvant phase, analyses were segmented into 2 treatment phases to manage the difficulties of analyzing the full treatment schedule comprehensively. Given that healthcare utilization was registered as count data, thus expected to be Poisson distributed, we employed log-linked generalized linear mixed models to test if different grades of thrombocytopenia were associated with unplanned hospital interactions during both treatment phases. Additionally, this method was repeated with anemia, leukocytopenia, lymphocytopenia, and neutropenia as primary covariates for separate analyses. The repeated measures of myelotoxicity grades per patient were included as a fixed effect. Other covariates such as sex, the interaction between female sex and thrombocytopenia grades, repeated measures of tumor progression, concomitant use of PPI, seizure-modifying treatments, and concomitant corticosteroid use, extent of tumor resection, age at diagnosis, BSA, tumor location, temporal presence of vascular adverse events and whether a hospital interaction took place during the covid-19 pandemic were added to the model through forward selection if their addition resulted in a decreased AIC of at least 5 points. Additionally, a random intercept variable was added to account for inter-patient variability. To further explore the association between thrombocytopenia and specific types of healthcare utilization, log-linked generalized linear models were fit for ER presentations and the number of days spent admitted to the hospital during both treatment phases. During the adjuvant phase, outcomes were normalized using the number of started adjuvant courses to correct for the effect of discontinued treatment schedules. For statistical analyses, the significance level was set at .05. Data handling and demographic comparisons were done using the Pandas and statsmodels libraries using Python version 3.12, and the fitting of both generalized linear mixed models and generalized linear models was done in IBM SPSS version 27.0.^24–27^

## Results

### Study Design and Patient Characteristics

**Figure 1.**
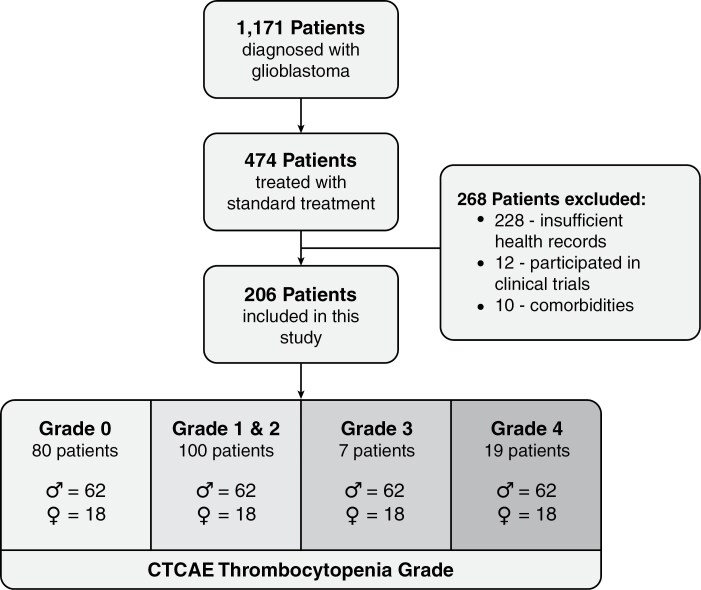
Patient cohort flowchart.

Among 474 eligible patients, 228 (48.1%) had complete electronic patient records available. Twelve (2.5%) patients were excluded from our study due to participation in an experimental trial, and 10 (2.1%) patients were excluded due to receiving concomitant treatment for unrelated disorders influencing healthcare utilization, resulting in the inclusion of 206 (43.5%) patients (Figure 1). The median age of our population was 58.0 (IQR = 48.5–66.0) years, and 35.9% (74/206) of patients were female (Table 1). Females had lower BSA (mean: 1.87, SD: 0.19) than males (mean: 2.1, SD: 0.18), *P* < .001, and had a lower median age at diagnosis (median = 55.9, IQR: 45.2–64.6) than males (median = 59.7, IQR: 50.2–66.2 years), *P* = .022.

**Table 1.** Patient Demographics, Stratified Per Nadir CTCAE Thrombocytopenia Grade of 206 Patients Treated With Maximal Safe Resection of the Tumor, 6 Weeks of Concomitant Chemo-radiotherapy and 6 Cycles of Adjuvant Temozolomide Chemotherapy

		Demographics per CTCAE thrombocytopenia grade				
	All patients	Grade 0	Grade 1	Grade 2	Grade 3	Grade 4
Total *n* patients, (%)	206	80 (38.9%)	86 (41.8%)	14 (6.8%)	7 (3.4%)	19 (9.2%)
Sex						
Male	132 (64.1)	62 (77.5)	56 (65.1)	6 (42.9)	3 (42.9)	5 (26.3)
Female	74 (35.9)	18 (22.5)	30 (34.9)	8 (57.1)	4 (57.1)	14 (73.7)
Age at diagnosis						
Median	58.0	56,1	58,6	56	51,9	61,8
* 25%–75% range*	[48.5, 66.0]	[48.0, 63.5]	[47.9, 67.0]	[48.9, 65.6]	[50.7, 55.8]	[57.9, 65.8]
BSA						
Mean	2.0	2.0	2.0	1.9	1.9	2.0
* Standard deviation*	0.2	0.2	0.2	0.2	0.3	0.2
Type of resection						
Complete/gross	36 (17.5)	11 (13.8)	17 (19.8)	3 (21.4)	2 (28.6)	3 (15.8)
Subtotal (≥90%)	53 (25.7)	29 (36.2)	14 (16.3)	5 (35.7)	1 (14.3)	4 (21.1)
Partial resection (<90%)	107 (52.0)	37 (46.2)	49 (57.0)	5 (35.7)	4 (57.1)	12 (63.2)
Biopsy	9 (4.4)	3 (3.8)	5 (5.8)	1 (7.1)	0 (0.0)	0 (0.0)
Missing	1 (0.5)	0 (0.0)	1(1.2)	0 (0.0)	0 (0.0)	0 (0.0)
Tumor location						
Frontal	79 (38.3)	30 (37.5)	29 (33.7)	5 (35.7)	3 (42.9)	12 (63.2)
Not frontal	127 (61.7)	50 (62.5)	57 (66.3)	9 (64.3)	4 (57.1)	7 (36.8)
Tumor side						
Right	105 (51.0)	43 (53.8)	35 (40.7)	7 (50.0)	4 (57.1)	16 (84.2)
Left	94 (45.6)	35 (43.8)	47 (54.7)	7 (50.0)	2 (28.6)	3 (15.8)
Bilateral	7 (3.4)	2 (2.5)	4 (4.7)	0 (0.0)	1 (14.3)	0 (0.0)
KPS before resection						
<70%	9 (4.3)	4 (5.0)	1 (1.2)	1 (7.1)	0 (0.0)	3 (15.8)
≥70%	145 (70.4)	52 (65.0)	63 (73.3)	10 (71.4)	6 (85.7)	14 (73.7)
Missing	52 (25.2)	24 (30.0)	22 (25.6)	3 (2.14)	1 (14.3)	2 (10.5)
KPS before CRT						
<70%	3 (1.5)	2 (2.5)	0 (0.0)	0 (0.0)	0 (0.0)	1 (5.3)
≥70%	141 (68.4)	54 (67.5)	60 (69.8)	9 (64.3)	6 (85.7)	12 (63.2)
Missing	62 (30.1)	24 (30.0)	26 (30.2)	5 (35.7)	1 (14.3)	6 (31.6)
KPS before ADJ						
<70%	2 (1.0)	2 (2.5)	0 (0.0)	0 (0.0)	0 (0.0)	0 (0.0)
≥70%	122 (59.2)	47 (58.8)	48 (50.0)	11 (78.6)	6 (85.7)	10 (52.6)
Missing	82 (39.8)	33 (41.3)	48 (50.0)	3 (21.4)	1 (14.2)	9 (47.4)
Adjuvant courses started						
0	8 (3.9)	1 (1.3)	1 (1.2)	0 (0.0)	1 (14.3)	5 (26.3)
1	8 (3.9)	4 (5.0)	0 (0.0)	1 (7.1)	0 (0.0)	3 (15.8)
2	8 (3.9)	1 (1.1)	2 (2.3)	3 (21.4)	1 (14.3)	1 (5.3)
3	12 (5.8)	5 (6.3)	5 (5.8)	1 (7.1)	0 (0.0)	1 (5.3)
4	11 (5.3)	5 (6.3)	5 (5.8)	0 (0.0)	0 (0.0)	1 (5.3)
5	15 (7.3)	7 (8.8)	7 (8.1)	1 (7.1)	0 (0.0)	0 (0.0)
6	144 (69.9)	57 (71.3)	66 (76.7)	8 (57.1)	5 (71.4)	8 (42.1)
Corticosteroids CRT						
Yes	93 (45.1)	39 (48.8)	37 (43.0)	7 (50.0)	2 (28.6)	8 (42.1)
No	113 (54.9)	41 (51.2)	49 (57.0)	7 (50.0)	5 (71.4)	11 (57.9)
PPI CRT						
Yes	82 (39.8)	32 (40.0)	34 (39.5)	4 (28.6)	1 (14.3)	11 (57.9)
No	124 (60.2)	48 (60.0)	52 (60.5)	10 (71.4)	6 (85.7)	8 (42.1)
Seizure-modifying treatment CRT						
Yes	93 (45.1)	39 (48.8)	37 (43.0)	7 (50.0)	2 (28.6)	8 (42.1)
No	113 (54.9)	41 (51.2)	49 (57.0)	7 (50.0)	5 (71.4)	11 (57.9)
Corticosteroids ADJ						
Yes	90 (43.7)	32 (40.0)	37 (43.0)	7 (50.0)	3 (42.9)	11 (57.9)
No	115 (55.8)	47 (60.0)	49 (57.0)	7 (50.0)	4 (57.1)	8 (42.1)
Missing	1 (0.5)	1 (1.3)	0 (0.0)	0 (0.0)	0 (0.0)	0 (0.0)
PPI ADJ						
Yes	94 (45.6)	35 (43.8)	37 (43.0)	7 (50.0)	4 (57.1)	11 (57.9)
No	111 (53.9)	44 (55.0)	49 (57.0)	7 (50.0)	3 (42.9)	8 (42.1)
Missing	1 (0.5)	1 (1.3)	0 (0.0)	0 (0.0)	0 (0.0)	0 (0.0)
Seizure-modifying treatment ADJ						
Yes	107 (51.9)	46 (57.5)	41 (47.7)	10 (71.4)	2 (28.6)	8 (42.1)
No	98 (47.6)	33 (41.3)	45 (52.3)	4 (28.6)	5 (71.4)	11 (57.9)
Missing	1 (0.5)	1 (1.3)	0 (0.0)	0 (0.0)	0 (0.0)	0 (0.0)
Vascular adverse event						
None	185 (89.8)	75 (0.94)	81 (94.2)	10 (71.4)	5 (71.4)	17 (89.5)
Cerebral hemorrhage	6 (2.9)	2 (2.5)	2 (2.3)	1 (7.1)	0 (0.0)	0 (0.0)
Deep vein thrombosis	5 (2.4)	1 (1.3)	1 (1.2)	1 (7.1)	1 (14.3)	1 (5.3)
Ischemic stroke	3 (1.5)	1 (1.3)	1 (1.2)	1 (7.1)	0 (0.0)	0 (0.0)
Pulmonary embolism	3 (1.5)	0 (0.0)	1 (1.2)	1 (7.1)	0 (0.0)	1 (5.3)
Cerebral sinus thrombosis	1 (0.5)	0 (0.0)	0 (0.0)	0 (0.0)	1 (14.3)	0 (0.0)
Treated during Covid-19						
No	189 (91.7)	71 (88.8)	78 (90.7)	14 (100.0)	7 (100.0)	19 (100.0)
Yes	17 (8.3)	9 (11.2)	8 (9.3)	0 (0.0)	0 (0.0)	0 (0.0)

Abbreviations: CRT, chemo-radiotherapy treatment phase; ADJ, adjuvant treatment phase; BSA, body surface area; KPS, Karnofsky performance score; PPI, Proton-pump inhibitors.

### Higher Grades of Thrombocytopenia are More Prevalent in Females

Of 206 patients, thrombocytopenia of any severity occurred in 126 (61.1%) patients; 86 (41.8%) developed grade 1, 14 (6.8%) developed grade 2, 7 (3.4%) grade 3, and 19 (9.2%) grade 4 thrombocytopenia (Table 1). Supplementary Table 1 provides an overview of the occurrence of all grades of thrombocytopenia per treatment phase, as stratified by sex. Thrombocytopenia grades differed between sexes (*P* < .001), with grade 4 thrombocytopenia developing in 18.9% (14/74) of females and 3.8% (5/132) of males. Higher thrombocytopenia grades were associated with fewer started treatment cycles (*P* < .001), 71.3% (57/80) of patients without thrombocytopenia started 6 cycles of adjuvant temozolomide, whereas only 42.1% (8/19) of patients who developed grade 4 thrombocytopenia started their sixth cycle. A nadir thrombocyte count of <50 000/µL (CTCAE grade 3 or 4) occurred in 9.6% and 9.4% of patients during the CRT and adjuvant phase respectively and did not differ in duration between females and males (median = 29.0 days, IQR = 13.5–43.5 vs. median = 28.0 days, IQR = 12.0–38.0, respectively, *P* = .640).

Lymphocytopenia was the most common form of myelotoxicity observed throughout the entire treatment, with 63.6% of the cohort (110/185) affected, a pattern consistent in both the CRT (58.3%) and adjuvant (75.9%) treatment phases. In contrast, neutropenia was the least common, with 36.9% (69/199) of patients experiencing it during treatment. Among severe myelotoxicities (CTCAE grade 3–4), leukocytopenia was the most prevalent, affecting 22.3% (46/202) of patients across the treatment timeline. This was also the most common severe myelotoxicity during CRT (11.7%, 31/187) and adjuvant treatment (15.3%, 31/203). Supplementary Table 3 provides a detailed overview of myelotoxicity types and their occurrence throughout the treatment phases.

Thirteen patients (6.3%) received a total of 33 transfusions in our cohort, of which 10 (4.9%) were female and 3 (1.5%) were male. All received transfusions were at a nadir thrombocyte count of <25 000/µL. The median number of transfusions among these patients was 2 (IQR: 1–4, and a maximum of 6 transfusions). The median number of transfusions did not differ between female and male patients who developed grade 4 thrombocytopenia, with a median number of transfusions in females of 2.0 (IQR: 1.0–4.0) and 1.0 (IQR: 1.0–3.0) in males, *P* = .39.

#### Grade 4 thrombocytopenia is associated with increased healthcare utilization during the CRT phase and is the largest in females

**Figure 2.**
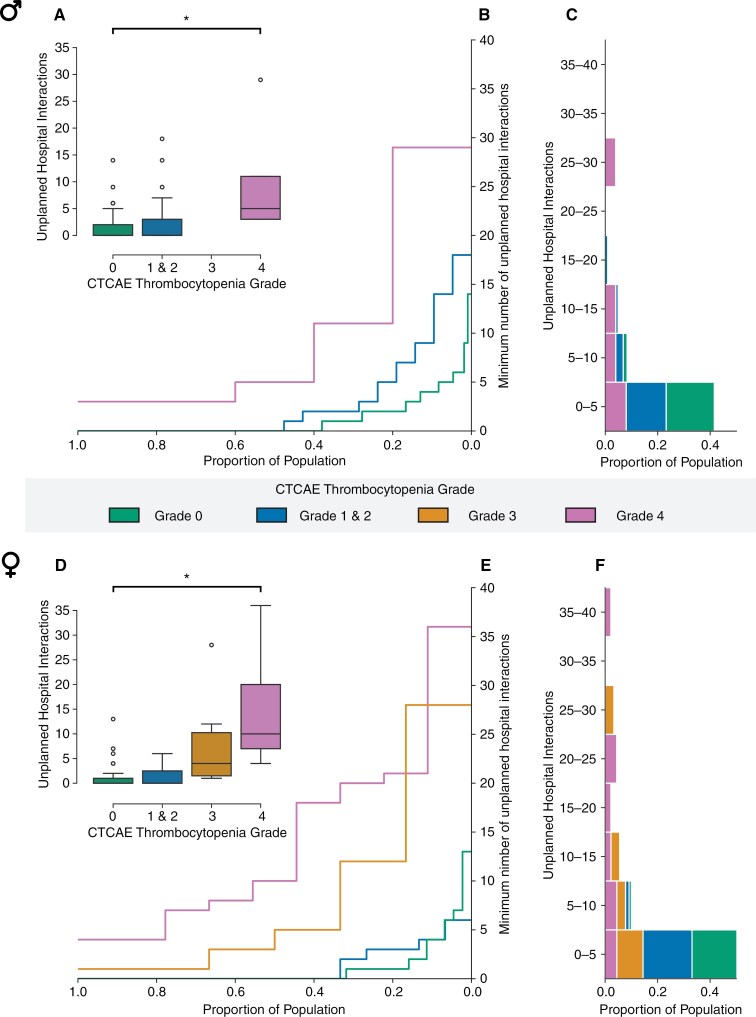
Grade 4 thrombocytopenia is associated with increased unplanned hospital interactions during CRT with a disproportional increase in females. (A) Boxplot showing the median number of unplanned hospital interactions per CTCAE thrombocytopenia grade in male patients. (B) ECDF-plot showing the minimum number of unplanned hospital interactions per fraction of the male patient population. (C) Histogram of unplanned hospital interactions per CTCAE thrombocytopenia grade in male patients. (D) Boxplot showing the median number of unplanned hospital interactions per CTCAE thrombocytopenia grade in female patients. (E) ECDF-plot showing the minimum number of unplanned hospital interactions per fraction of the female patient population. (F) Histogram of unplanned hospital interactions per CTCAE thrombocytopenia grade in female patients. Abbreviations: Prop: proportion of population, CTCAE: Common Terminology Criteria for Adverse Events.

Based on our hypothesis, we expected that the development of higher grades of thrombocytopenia during the CRT phase would lead to an increase in unplanned hospital interactions. The results confirmed this hypothesis, showing a significant increase in hospital interactions when grade 4 thrombocytopenia developed in comparison to patients who did not develop thrombocytopenia (OR = 17.4, 95% CI = 9.1–33.2, *P* < .001, Figure 2 and Table 2). These findings indicate that patients who developed grade 4 thrombocytopenia during the CRT phase had substantially more unplanned hospital interactions in comparison to patients who did not develop thrombocytopenia. The effect of grade 4 thrombocytopenia on healthcare utilization was moderated by sex (*P* = .033). Corrected analyses showed that the occurrence of grade 4 thrombocytopenia, had a stronger positive effect on healthcare utilization in females than in males during the CRT phase: females who developed grade 4 thrombocytopenia experienced a larger number of unplanned hospital interactions in comparison to males (grade 4 thrombocytopenia compared to no thrombocytopenia, OR = 5.9 in females vs OR = 4.4 in males). On average, females had 32.6% more unplanned hospital interactions per week (mean: 7.5, SD: 5.0) than males (mean: 5.6, SD: 3.9) during the CRT weeks when grade 4 thrombocytopenia was present. Unplanned healthcare utilization during CRT primarily consisted of tests (blood drawls) and hospitalization days. Supplementary Table 4 provides an overview of the specific types of unplanned healthcare resources utilized.

**Table 2.** Results of the Generalized Linear Mixed Model Examining the Association Between the Severity of Thrombocytopenia and Unplanned Healthcare Utilization During CRT Phase

	Exp. Coeff.	t	Sig.	Exp. 95% confidence interval	
				2.5%	97.5%
*Intercept*	0.25	−1.3	.20	0.031	2.1
*Thrombocytopenia*					
Grade 0	—	—	—	—	—
Grades 1 and 2	2.3	1.7	.082	0.90	5.8
Grade 3	5.2	1.2	.22	0.36	73.5
Grade 4	17.4	8.7	<.001	9.1	33.2
*Sex*					
Male	—	—	—	—	—
Female	0.47	−2.2	.027	0.24	0.92
*Interaction female sex by grade thrombocytopenia*					
Grade 0	—	—	—	—	—
Grades 1 and 2	1.2	0.24	.81	0.27	5.3
Grade 3	6.6	1.3	.18	0.41	104.9
Grade 4	2.8	2.1	.033	1.1	7.4
*BSA*	0.87	−0.27	.78	0.33	2.3
*Corticosteroids*					
No	—	—	—	—	—
Yes	1.4	1.6	.12	0.92	2.0
*Tumor side*					
Left	—	—	—	—	—
Right	1.7	2.3	.022	1.1	2.6

Link type: Log-Link

Development of lower grades of thrombocytopenia (grades 1–3) was not associated with increased unplanned hospital interactions compared to patients who did not develop thrombocytopenia. For males, grades 1 and 2 had an OR of 2.3 (*P* = .082), and grade 3 had an OR of 5.2 (*P* = .22). For females, grades 1 and 2 had an OR of 4.0 (*P* = .082), and grade 3 had an OR of 12.3 (*P* = .18). These results indicate that neither the grades of thrombocytopenia nor the modifying effect of the female sex were significantly associated with hospital interactions. Furthermore, a right-sided tumor was associated with increased unplanned hospital interactions in comparison to patients having a left-sided tumor (OR = 1.7, 95% CI = 1.1–2.6, *P* = .022).

The development grade 4 leukocytopenia (OR = 9.0, 95% CI = 4.2–19.3, *P* < .001, significant interaction with female sex), lymphocytopenia (OR = 22.6, 95% CI = 3.0–131.6, *P* < .001, no interaction with female sex) and neutropenia (OR = 18.9, 95% CI = 6.7–52.8, *P* < .001, no interaction with female sex) showed a significant association with increased healthcare utilization compared to no development of their respective myelotoxicity. As for the development of grade 3 myelotoxicity, neutropenia alone was associated with increased unplanned healthcare utilization (OR = 44.8, 95% CI = 15.5–129.2, *P* < .001, no interaction with female sex). Results of the examined association between different types of myelotoxicity and unplanned healthcare utilization during the CRT phase are presented in Supplementary Table 5.

#### The onset of thrombocytopenia is associated with increased healthcare utilization during the ADJ phase and is the largest in grade 4 thrombocytopenia

**Figure 3.**
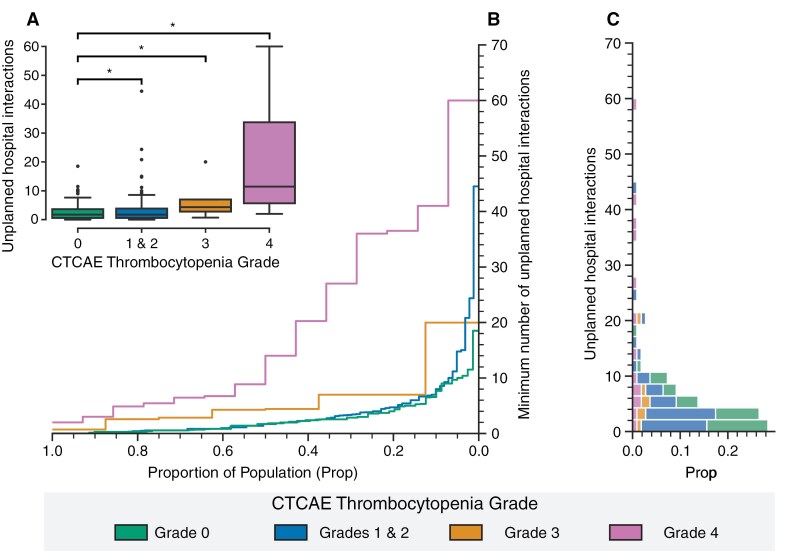
The occurrence of thrombocytopenia is associated with increased unplanned hospital interactions during adjuvant treatment. (A) Boxplot showing the median number of unplanned hospital interactions per CTCAE thrombocytopenia grade. (B) ECDF-plot showing the minimum number of unplanned hospital interactions per fraction of the patient population. (C) Histogram of unplanned hospital interactions per CTCAE thrombocytopenia grade. Abbreviations: Prop: proportion of population, CTCAE: Common Terminology Criteria for Adverse Events.

During the adjuvant phase of treatment, our model showed consistent findings regarding the effect of thrombocytopenia on healthcare utilization in comparison to the CRT phase. Patients who developed thrombocytopenia had increased unplanned hospital interactions during the adjuvant phase, with the largest increases in grade 4 (OR = 6.5, 95% CI = 4.6–9.1, *P* < .001) and grade 3 (OR = 2.3, 95% CI = 1.4–4.0, *P* = .002), and a minor increase in patients with grades 1 and 2 (OR = 1.4, 95% CI = 1.1–1.7, *P* = .003, Table 3 and Figure 3) in comparison to patients who did not develop thrombocytopenia. Female sex was not an effect moderator of the relation between grade 4 thrombocytopenia and unplanned hospital interactions. During months when patients experienced grade 4 thrombocytopenia, male patients had an average of 26.6 (SD: 9.6) monthly unplanned hospital interactions, while female patients averaged 26.6 (SD: 16.6). The most utilized resources included outpatient clinic visits, blood tests, and phone consultations with medical specialists (Supplementary Table 4).

**Table 3.** Results of the Generalized Linear Mixed Model Examining the Association Between the Severity of Thrombocytopenia and Unplanned Healthcare Utilization During the Adjuvant Treatment Phase

	Exp. Coeff.	t	Sig.	Exp. 95% confidence interval	
				2.5%	97.5%
*Intercept*	1.2	0.32	.75	0.44	3.1
*Thrombocytopenia*					
Grade 0	—	—	—	—	—
Grades 1 and 2	1.4	2.9	.003	1.1	1.7
Grade 3	2.3	3.1	.002	1.4	4.0
Grade 4	6.5	10.7	<.001	4.6	9.1
*Progression of disease*					
No	—	—	—	—	—
Yes	2.6	8.0	<.001	2.0	3.2
*Seizure-modifying treatment*					
No	—	—	—	—	—
Yes	1.3	2.7	.007	1.1	1.6
*Corticosteroids*					
No	—	—	—	—	—
Yes	1.7	4.7	<.001	1.4	2.1
*Proton pump inhibitors*					
No	—	—	—	—	—
Yes	0.87	−1.3	.18	0.70	1.1
*Tumor side*					
Left	—	—	—	—	—
Right	1.6	4.4	<.001	1.3	1.9
*Extend of resection*					
Biopsy	—	—	—	—	—
Partial	1.1	0.35	.73	0.69	1.7
Subtotal	0.77	−1.0	.30	0.47	1.3
Gross total	0.62	−1.8	.07	0.37	1.0
Link type: Log-Link					

Additionally, patients who had tumor progression (OR = 2.6, 95% CI [2.0–3.2], *P* < .001), received concomitant seizure-modifying treatment (OR = 1.3, 95% CI [1.1–1.6], *P* = .007), or used concomitant corticosteroids compared to those who did not (OR = 1.7, 95% CI [1.3–2.1], *P* < .001) during the adjuvant treatment phase had increased unplanned hospital interactions. Similarly, patients with a right-sided tumor in comparison to patients who had a left-sided tumor (OR = 1.6, 95% CI = 1.3–1.9, *P* < .001) also had increased hospital interactions.

For Leukocytopenia (OR = 16.1, 95%-CI = 3.1–82.1, *P* < .001), lymphocytopenia (OR = 3.9, 95% CI = 1.1 13.9, *P* = .03), and neutropenia (OR = 4.5, 95%-CI = 2.7–7.6, *P* < .001) development of grade 4 myelotoxicity was associated with increased healthcare utilization in comparison to no those who did not develop myelotoxicity. Additionally, the development of both grade 3 lymphocytopenia (OR = 7.0, 95% CI = 3.5–14.3, *P* < .001) and neutropenia (OR = 2.0, 95% CI = 1.5–2.8, *P* < .001) positively associated with increased healthcare utilization in comparison to those who did not develop their respective myelotoxicity.

#### Thrombocytopenia is linked to higher hospitalization rates and duration and is most pronounced in females

A total of 77 hospital admissions occurred in our cohort; 25 (32.5%) hospital admissions were in patients without any grade of thrombocytopenia and 26 (33.8%) in patients who developed severe thrombocytopenia (grade 3 or 4). Hospital admissions occurred in 46 (22.3%) patients, of whom 23 (50.0%) were females.

During the CRT phase, hospital admissions were prolonged among patients developing grade 1–3 thrombocytopenia in comparison to patients who did not develop thrombocytopenia (grade 1 and 2: OR = 3.3, 95% CI = 3.5–14.8, *P* < .001 and grade 3: OR = 3.0, 95% CI = 1.4–6.3, *P* < .005, respectively), whereas no association was found for the development of grade 4 thrombocytopenia in comparison to no thrombocytopenia (OR = 2.5, 95% CI = 0.59–10.8, *P* = 0.21, Supplementary Table 2). However, the effect of grades 4 and 3 thrombocytopenia on the number of days spent hospitalized was positively modified by female sex (OR = 7.2, 95% CI = 3.5–14.8, *P* < .001 and OR = 2.3, 95% CI = 2.8–162.7, *P* = .003), indicating that the observed association between grade 4 and 3 thrombocytopenia on days spent hospitalized in comparison to patients who did not develop thrombocytopenia is largest in females. Male patients who developed grade 4 thrombocytopenia had an average of 0.8 days (SD: 0.8) spent in the hospital during CRT, whereas female patients had an average of 4.4 days (SD: 5.3) spent in the hospital during CRT. Additionally, corrected for the effect of thrombocytopenia, a right-sided tumor was associated with an increased number of days spent hospitalized in comparison to a left-sided tumor (OR = 6.2, 95% CI = 2.8–13.6, *P* < .001).

During the adjuvant phase, the development of grade 4 and grades 1 and 2 thrombocytopenia in comparison to no development of thrombocytopenia were associated with increased number of days spent admitted to the hospital (OR = 3.1, 95% CI = 1.6–6.1, *P* = .001, OR = 5.5, 95% CI = 3.1–9.9, *P* < .001, respectively, Supplementary Table 3). Patients who developed grade 4 thrombocytopenia had a higher mean number of days spent in the hospital than patients who did not develop thrombocytopenia (mean: 4.6, SD: 6.1, vs. mean: 1.2, SD: 3.7, respectively). The effect of grade 3 thrombocytopenia was positively modified by female sex (OR = 5.4, 95% CI = 2.8–10.4, *P* < .001), indicating that females with grade 3 thrombocytopenia had a higher number of days spent admitted to the hospital compared to males with the same condition. Conversely, the effect of grades 1 and 2 thrombocytopenia was negatively modified by female sex (OR = 0.19, 95% CI = 0.089–0.41, *P* < .001), suggesting that females with grades 1 and 2 thrombocytopenia had fewer hospital admissions and shorter stays compared to their male counterparts.

#### Severe thrombocytopenia is not associated with an increase in emergency room presentations

In total, 151 ER presentations were registered in 93 (45.1%) patients, of which 71 (47.02%) presentations were by patients without thrombocytopenia and 28 (18.54%) presentations by patients who developed severe thrombocytopenia (grades 3–4). Of all 93 patients who had ER presentations 38 (40.9%) were female.

Patients who developed grade 4 thrombocytopenia in comparison to patients who did not develop thrombocytopenia had increased ER presentations during the CRT phase (OR = 4.3, 95% CI = 2.0–9.2, *P* < .001, Supplementary Table 4) but not during the ADJ phase (OR = 2.3, 95% CI = 0.9–5.6, *P* = .076, Supplementary Table 5). During CRT, patients who developed grade 4 thrombocytopenia had a mean of 0.6 ER presentations (SD: 0.7), whereas patients who did not develop thrombocytopenia had a mean of 0.2 ER presentations (SD: 0.4). During the ADJ phase, the development of tumor progression was positively associated with an increased number of ER presentations in comparison to months where tumor progression did not occur in all patients (OR = 4.1, 95% CI = 2.2–7.5, *P* < .001).

## Discussion

In this retrospective cohort study, we systematically reviewed patient health records to quantify the utilization of unplanned healthcare resources and to investigate its association with thrombocytopenia in patients treated for glioblastoma. We found that more than half of our included patients who received maximal safe resection, 6 weeks of concomitant chemo-radiotherapy, and 6 cycles of adjuvant temozolomide chemotherapy for glioblastoma develop thrombocytopenia and we demonstrate that patients who develop grade 4 thrombocytopenia at any stage of their treatment exhibit a disproportionate increase in healthcare utilization. Among females, the described association is most pronounced, primarily if thrombocytopenia occurs during CRT.

The findings of this study aligned with our hypothesis, we expected higher grades of thrombocytopenia to be associated with increased healthcare utilization as it can lead to severe bleeding and necessitates careful monitoring and early intervention.^10^ Increased healthcare utilization due to myelotoxicity, including thrombocytopenia, has been shown to raise the direct costs of care, sometimes by as much as 60%, highlighting the economic impact of managing these complications.^19,28^ Interestingly, females who developed grade 4 thrombocytopenia utilized more unplanned healthcare resources than their male counterparts during CRT. Similar sex-based disparities have been reported in lung cancer studies, where this discrepancy was not attributed to women receiving more treatment, but to men seeking less healthcare. This behavior was thought to contribute to the higher morbidity and mortality rates observed in men.^29^ The absence of a sex-based disparity in healthcare utilization during the adjuvant treatment phase could be due to the smaller number of events or may indicate a lack of sex modification in this phase, warranting further investigation. Other factors associated with increased healthcare utilization included the presence of a right-sided tumor during the CRT and adjuvant phases, along with the concurrent use of seizure-modifying treatments and corticosteroids during the adjuvant phase. The finding of increased healthcare utilization in patients with right-sided tumors is unexpected, given that left-sided tumors are typically located in eloquent brain regions and are often subject to less thorough resections, which were expected to result in greater healthcare utilization. At present, the underlying reasons for this observed result remain unclear, and the possibility of this finding being due to chance cannot be excluded.^30^ The increased healthcare utilization associated with concomitant seizure-modifying treatments and corticosteroids during the adjuvant phase is expected, as these treatments are commonly prescribed to manage glioblastoma-associated neurological symptoms. We speculate that these corresponding symptoms necessitate additional healthcare resources.

We found that more than half of the patients developed some degree of thrombocytopenia during standard treatment. Although most patients experienced only moderate thrombocytopenia, more than ten percent developed severe thrombocytopenia. The incidence of severe thrombocytopenia was comparable during both the CRT and adjuvant phases but higher than reported in clinical trials, where severe thrombocytopenia developed in five percent of patients enrolled in five clinical trials.^31^ Patient selection for clinical trials may skew towards patients who are not fully representative of clinical practice, as patient selection might result in younger individuals with a more favorable clinical condition.^32^ This is further supported by the higher occurrences of severe myelotoxicity in extensive population-based retrospective cohorts.^15,20^ The findings of these studies align with ours, emphasizing the differences in the occurrence of thrombocytopenia between population-based studies and selected patient groups. The frequencies of both moderate and severe thrombocytopenia are often misrepresented in clinical trials compared to population-based cohorts, with clinical practice populations showing higher occurrences.

Consistent with prior studies, females were more likely to develop grade 4 thrombocytopenia than males, although the duration of the condition was similar across both sexes.^15,20,31,33^ Similar sex differences have been found for other indications and chemotherapies.^34^ It is likely that the observed differences are due to the differing pharmacokinetics and pharmacodynamics of temozolomide in males and females. Sex and BSA independently influence the clearance of oral temozolomide, males have a faster temozolomide clearance than females and patients with a higher BSA have a higher clearance than those with lower BSA.^16,17^ At present, the temozolomide dose is merely based on BSA, thus ignoring sex-specific pharmacokinetics. When myelotoxicity occurs, most patients have treatment interruptions, and a large proportion of patients stop treatment.^15,35,36^ We also observed that patients with severe thrombocytopenia received fewer courses of temozolomide, indicating that females specifically are more prone to treatment adjustments or treatment discontinuation. Early discontinuation of first-line treatment has been linked to reduced overall survival in women, suggesting that adverse events leading to treatment cessation may affect outcomes.^15^ The influence of early termination of first-line treatment on patient prognosis; however, was beyond the scope of this study and was not analyzed.

This study has several strengths. We chose an approach that represents daily practice within a large academic brain tumor center. We highlighted the impact of thrombocytopenia on unplanned healthcare utilization. There are also limitations to this study. Despite a thorough review of health records for patient demographics, glioblastoma-related symptoms like epileptic seizures were not extracted. It is unlikely that these symptoms or unrecorded adverse events were present during the occurrence of thrombocytopenia, but it cannot be fully excluded. Additionally, the relationship between healthcare utilization and the quality of life experienced by the patient is intricate. At present, we cannot infer the effects of healthcare utilization on the quality of life of patients treated with standard treatment. Such a topic would optimally be studied in a dedicated prospective trial. We did not include marital status in our study due to the inconsistent availability of this data. Epidemiological studies have shown an independent association between marital status and healthcare utilization, suggesting that this factor could have influenced our findings.^37,38^ We believe the absence of marital status is not a major limitation, as a sufficient social support system—often provided by a partner—is a prerequisite for treatment at the cohort of its institution. Additionally, due to less detailed reporting of healthcare resource utilization in the earlier years of our cohort, many patients were excluded, resulting in a smaller population that is disproportionately treated in the later years of our cohort. Although this disproportion exists, we do not expect it to have introduced bias into our results, as treatment protocols remained consistent throughout the cohort period, we do expect that the smaller population size may have prevented us from identifying potential expected associations, particularly during CRT with hospital admissions and ER presentations.

In conclusion, we show increased healthcare utilization in patients developing thrombocytopenia during the standard treatment for glioblastoma, with a disproportional increase in females who developed higher grades of thrombocytopenia. Additionally, as females develop severe myelotoxicity more frequently, future research warrants the evaluation of sex differences in pharmacokinetics, pathophysiology, and health perception, and further optimization of treatment to decrease unplanned healthcare utilization.

## Supplementary material

Supplementary material is available online at *Neuro-Oncology Practice* (https://academic.oup.com/nop/).

npaf013_suppl_Supplementary_Materials

## Data Availability

Data will be made available upon reasonable request.

## Declarations

This manuscript has previously been submitted to Neuro-Oncology. All coauthors have read and approved the manuscript submission to this Journal. I agree to the full-color reproduction of the manuscript.
