# Supplementary material for: Severe temozolomide-induced thrombocytopenia is linked to increased healthcare utilization in glioblastoma and disproportionally impacts female patients
Source: Neurooncol Pract. 2025 Jan 22;12(4):678–90. doi: 10.1093/nop/npaf013 (PMC12349765; doi:10.1093/nop/npaf013)
Supplement: npaf013_suppl_Supplementary_Materials [file npaf013_suppl_supplementary_materials.docx]

## 9. Appendix

### 9.1 Supplementary tables:

**Supplementary Table 1:** Patient demographics, stratified per nadir CTCAE thrombocytopenia grade of 206 patients treated with maximal safe resection of the tumour, six weeks of concomitant chemoradiotherapy and six cycles of adjuvant temozolomide chemotherapy.

|  |  | **Demographics per CTCAE thrombocytopenia grade** | | | | |  |
| --- | --- | --- | --- | --- | --- | --- | --- |
|  | All patients | Grade 0 | Grade 1 | Grade 2 | Grade 3 | Grade 4 | Comparison |
| Total n. patients, (%) | 206 | 80 (38.9%) | 86 (41.8%) | 14 (6.8%) | 7 (3.4%) | 19 (9.2%) |  |
|  |  |  |  |  |  |  |  |
| Sex |  |  |  |  |  |  | Fisher’s Exact Test |
| Male | 132 (64.1) | 62 (77.5) | 56 (65.1) | 6 (42.9) | 3 (42.9) | 5 (26.3) | F = 21.938, p < .001 |
| Female | 74 (35.9) | 18 (22.5) | 30 (34.9) | 8 (57.1) | 4 (57.1) | 14 (73.7) |  |
|  |  |  |  |  |  |  |  |
| Age at diagnosis |  |  |  |  |  |  | Kruskal-Wallis |
| Median | 58.0 | 56,1 | 58,6 | 56 | 51,9 | 61,8 | U = 3.866, p = .424 |
| *25-75% range* | [48.5, 66.0] | [48.0, 63.5] | [47.9, 67.0] | [48.9, 65.6] | [50.7, 55.8] | [57.9, 65.8] |  |
|  |  |  |  |  |  |  |  |
| BSA |  |  |  |  |  |  | One-Way ANOVA |
| Mean | 2.0 | 2.0 | 2.0 | 1.9 | 1.9 | 2.0 | F = 1.659, p = .161 |
| *Standard deviation* | 0.2 | 0.2 | 0.2 | 0.2 | 0.3 | 0.2 |  |
|  |  |  |  |  |  |  |  |
| Type of resection |  |  |  |  |  |  | Fisher’s Exact Test |
| Complete/gross | 36 (17.5) | 11 (13.8) | 17 (19.8) | 3 (21.4) | 2 (28.6) | 3 (15.8) | F = 13.050, p = .290 |
| Subtotal (≥90%) | 53 (25.7) | 29 (36.2) | 14 (16.3) | 5 (35.7) | 1 (14.3) | 4 (21.1) |  |
| Partial resection (<90%) | 107 (52.0) | 37 (46.2) | 49 (57.0) | 5 (35.7) | 4 (57.1) | 12 (63.2) |  |
| Biopsy | 9 (4.4) | 3 (3.8) | 5 (5.8) | 1 (7.1) | 0 (0.0) | 0 (0.0) |  |
| Missing | 1 (0.5) | 0 (0.0) | 1(1.2) | 0 (0.0) | 0 (0.0) | 0 (0.0) |  |
|  |  |  |  |  |  |  |  |
| Tumour location |  |  |  |  |  |  | Fisher’s Exact Test |
| Frontal | 79 (38.3) | 30 (37.5) | 29 (33.7) | 5 (35.7) | 3 (42.9) | 12 (63.2) | F = 5.955, p = .196 |
| Not frontal | 127 (61.7) | 50 (62.5) | 57 (66.3) | 9 (64.3) | 4 (57.1) | 7 (36.8) |  |
|  |  |  |  |  |  |  |  |
| Tumour side |  |  |  |  |  |  | Fisher’s Exact Test |
|  | 105 (51.0) | 43 (53.8) | 35 (40.7) | 7 (50.0) | 4 (57.1) | 16 (84.2) | F = 15.561, p = .029 |
| Right |  |  |  |  |  |  |  |
| Left | 94 (45.6) | 35 (43.8) | 47 (54.7) | 7 (50.0) | 2 (28.6) | 3 (15.8) |  |
| Bilateral | 7 (3.4) | 2 (2.5) | 4 (4.7) | 0 (0.0) | 1 (14.3) | 0 (0.0) |  |
|  |  |  |  |  |  |  |  |
| KPS before resection |  |  |  |  |  |  | Fisher’s Exact Test |
| <70% | 9 (4.3) | 4 (5.0) | 1 (1.2) | 1 (7.1) | 0 (0.0) | 3 (15.8) | F = 12.326, p = .069 |
| ≥70% | 145 (70.4) | 52 (65.0) | 63 (73.3) | 10 (71.4) | 6 (85.7) | 14 (73.7) |  |
| Missing | 52 (25.2) | 24 (30.0) | 22 (25.6) | 3 (2.14) | 1 (14.3) | 2 (10.5) |  |
|  |  |  |  |  |  |  |  |
| KPS before CRT |  |  |  |  |  |  | Fisher’s Exact Test |
| <70% | 3 (1.5) | 2 (2.5) | 0 (0.0) | 0 (0.0) | 0 (0.0) | 1 (5.3) | F = 5.050, p = .219 |
| ≥70% | 141 (68.4) | 54 (67.5) | 60 (69.8) | 9 (64.3) | 6 (85.7) | 12 (63.2) |  |
| Missing | 62 (30.1) | 24 (30.0) | 26 (30.2) | 5 (35.7) | 1 (14.3) | 6 (31.6) |  |
|  |  |  |  |  |  |  |  |
| KPS before ADJ |  |  |  |  |  |  | Fisher’s Exact Test |
| <70% | 2 (1.0) | 2 (2.5) | 0 (0.0) | 0 (0.0) | 0 (0.0) | 0 (0.0) | F = 8.783, p = 1.000 |
| ≥70% | 122 (59.2) | 47 (58.8) | 48 (50.0) | 11 (78.6) | 6 (85.7) | 10 (52.6) |  |
| Missing | 82 (39.8) | 33 (41.3) | 48 (50.0) | 3 (21.4) | 1 (14.2) | 9 (47.4) |  |
|  |  |  |  |  |  |  |  |
| Adjuvant courses started |  |  |  |  |  |  | Fisher’s Exact Test |
| 0 | 8 (3.9) | 1 (1.3) | 1 (1.2) | 0 (0.0) | 1 (14.3) | 5 (26.3) | F = 61.484, p < .001 |
| 1 | 8 (3.9) | 4 (5.0) | 0 (0.0) | 1 (7.1) | 0 (0.0) | 3 (15.8) |  |
| 2 | 8 (3.9) | 1 (1.1) | 2 (2.3) | 3 (21.4) | 1 (14.3) | 1 (5.3) |  |
| 3 | 12 (5.8) | 5 (6.3) | 5 (5.8) | 1 (7.1) | 0 (0.0) | 1 (5.3) |  |
| 4 | 11 (5.3) | 5 (6.3) | 5 (5.8) | 0 (0.0) | 0 (0.0) | 1 (5.3) |  |
| 5 | 15 (7.3) | 7 (8.8) | 7 (8.1) | 1 (7.1) | 0 (0.0) | 0 (0.0) |  |
| 6 | 144 (69.9) | 57 (71.3) | 66 (76.7) | 8 (57.1) | 5 (71.4) | 8 (42.1) |  |
|  |  |  |  |  |  |  |  |
| Corticosteroids CRT |  |  |  |  |  |  | Fisher’s Exact Test |
| Yes | 93 (45.1) | 39 (48.8) | 37 (43.0) | 7 (50.0) | 2 (28.6) | 8 (42.1) | F = 2.219, p = .705 |
| No | 113 (54.9) | 41 (51.2) | 49 (57.0) | 7 (50.0) | 5 (71.4) | 11 (57.9) |  |
|  |  |  |  |  |  |  |  |
| PPI CRT |  |  |  |  |  |  | Fisher’s Exact Test |
| Yes | 82 (39.8) | 32 (40.0) | 34 (39.5) | 4 (28.6) | 1 (14.3) | 11 (57.9) | F = 4.990, p = .286 |
| No | 124 (60.2) | 48 (60.0) | 52 (60.5) | 10 (71.4) | 6 (85.7) | 8 (42.1) |  |
|  |  |  |  |  |  |  |  |
| Seizure modifying treatment CRT |  |  |  |  |  |  | Fisher’s Exact Test |
| Yes | 93 (45.1) | 39 (48.8) | 37 (43.0) | 7 (50.0) | 2 (28.6) | 8 (42.1) | F = 1.586, p = .826 |
| No | 113 (54.9) | 41 (51.2) | 49 (57.0) | 7 (50.0) | 5 (71.4) | 11 (57.9) |  |
|  |  |  |  |  |  |  |  |
| Corticosteroids ADJ |  |  |  |  |  |  | Fisher’s Exact Test |
| Yes | 90 (43.7) | 32 (40.0) | 37 (43.0) | 7 (50.0) | 3 (42.9) | 11 (57.9) | F = 2.227, p = .712 |
| No | 115 (55.8) | 47 (60.0) | 49 (57.0) | 7 (50.0) | 4 (57.1) | 8 (42.1) |  |
| Missing | 1 (0.5) | 1 (1.3) | 0 (0.0) | 0 (0.0) | 0 (0.0) | 0 (0.0) |  |
|  |  |  |  |  |  |  |  |
| PPI ADJ |  |  |  |  |  |  | Fisher’s Exact Test |
| Yes | 94 (45.6) | 35 (43.8) | 37 (43.0) | 7 (50.0) | 4 (57.1) | 11 (57.9) | F = 2.017, p = .749 |
| No | 111 (53.9) | 44 (55.0) | 49 (57.0) | 7 (50.0) | 3 (42.9) | 8 (42.1) |  |
| Missing | 1 (0.5) | 1 (1.3) | 0 (0.0) | 0 (0.0) | 0 (0.0) | 0 (0.0) |  |
|  |  |  |  |  |  |  |  |
| Seizure modifying treatment ADJ |  |  |  |  |  |  | Fisher’s Exact Test |
| Yes | 107 (51.9) | 46 (57.5) | 41 (47.7) | 10 (71.4) | 2 (28.6) | 8 (42.1) | F = 6.125, p = .186 |
| No | 98 (47.6) | 33 (41.3) | 45 (52.3) | 4 (28.6) | 5 (71.4) | 11 (57.9) |  |
| Missing | 1 (0.5) | 1 (1.3) | 0 (0.0) | 0 (0.0) | 0 (0.0) | 0 (0.0) |  |
| Abbreviations: CRT, chemo-radiotherapy treatment phase; ADJ, adjuvant treatment phase; BSA, body surface area; KPS, Karnofsky performance score; PPI, Proton-pump inhibitors. | | | | | | | |

|  | **Entire treatment schedule** | | **CRT - phase** | | **ADJ - phase** | |
| --- | --- | --- | --- | --- | --- | --- |
| Patient sex | Male | Female | Male | Female | Male | Female |
| Grade 0 | 62 | 18 | 106 | 43 | 65 | 19 |
|  | *48.15%* | *25.33%* | *80.74%* | *59.46%* | *48.15%* | *27.94%* |
| Grade 1 | 56 | 30 | 17 | 14 | 56 | 30 |
|  | *41.48%* | *40.00%* | *12.59%* | *18.92%* | *41.48%* | *44.12%* |
| Grade 2 | 6 | 8 | 4 | 1 | 6 | 8 |
|  | *4.44%* | *10.67%* | *2.96%* | *1.35%* | *4.44%* | *4.41%* |
| Grade 3 | 3 | 4 | 0 | 6 | 3 | 3 |
|  | *2.22%* | *5.33%* | *0.00%* | *8.11%* | *2.22%* | *4.41%* |
| Grade 4 | 5 | 14 | 5 | 9 | 5 | 8 |
|  | *3.70%* | *18.67%* | *3.70%* | *12.16%* | *3.70%* | *11.76%* |
| Total | 132 | 74 | 132 | 74 | 135 | 68 |

**Supplementary Table 2:** Occurrences of thrombocytopenia grades per treatment phase, stratified by sex.

**Supplementary Table 3:** Occurrences of all included myelotoxicity grades per treatment phase.

|  | **Entire treatment** | | **CRT - phase** | | **ADJ - phase** | |
| --- | --- | --- | --- | --- | --- | --- |
| Myelotoxicity | n. | % | n. | % | n. | % |
| Population size | 206 | 100.0 | 206 | 100.0 | 203 | 100.0 |
| Thrombocytopenia | n. | % | n. | % | n. | % |
| Grade 0 | 81 | 39.3% | 151 | 73.3% | 84 | 41.4% |
| Grade 1 | 85 | 41.3% | 31 | 15.0% | 86 | 42.4% |
| Grade 2 | 14 | 6.8% | 5 | 2.4% | 14 | 6.9% |
| Grade 3 | 7 | 3.4% | 6 | 2.9% | 6 | 3.0% |
| Grade 4 | 19 | 9.2% | 13 | 6.3% | 13 | 6.4% |
| Missing | 0 | 0.0% | 0 | 0.0% | 0 | 0.0% |
| Anemia | n. | % | n. | % | n. | % |
| Grade 0 | 129 | 62.6% | 146 | 70.9% | 140 | 69.0% |
| Grade 1 | 48 | 23.3% | 32 | 15.5% | 37 | 18.2% |
| Grade 2 | 11 | 5.3% | 6 | 2.9% | 9 | 4.4% |
| Grade 3 | 14 | 6.8% | 3 | 1.5% | 13 | 6.4% |
| Grade 4 | 0 | 0.0% | 0 | 0.0% | 0 | 0.0% |
| Missing | 4 | 1.9% | 19 | 9.2% | 4 | 2.0% |
| Leukocytopenia | n. | % | n. | % | n. | % |
| Grade 0 | 91 | 44.2% | 134 | 65.0% | 105 | 51.7% |
| Grade 1 | 44 | 21.4% | 18 | 8.7% | 46 | 22.7% |
| Grade 2 | 21 | 10.2% | 11 | 5.3% | 17 | 8.4% |
| Grade 3 | 41 | 19.9% | 22 | 10.7% | 29 | 14.3% |
| Grade 4 | 5 | 2.4% | 2 | 1.0% | 2 | 1.0% |
| Missing | 4 | 1.9% | 19 | 9.2% | 4 | 2.0% |
| Lymphocytopenia | n. | % | n. | % | n. | % |
| Grade 0 | 75 | 36.4% | 86 | 41.7% | 53 | 26.1% |
| Grade 1 | 37 | 18.0% | 34 | 16.5% | 13 | 6.4% |
| Grade 2 | 51 | 24.8% | 45 | 21.8% | 20 | 9.9% |
| Grade 3 | 19 | 9.2% | 14 | 6.8% | 9 | 4.4% |
| Grade 4 | 3 | 1.5% | 1 | 0.5% | 1 | 0.5% |
| Missing | 21 | 10.2% | 26 | 12.6% | 107 | 52.7% |
| Neutropenia | n. | % | n. | % | n. | % |
| Grade 0 | 130 | 63.1% | 166 | 80.6% | 137 | 67.5% |
| Grade 1 | 40 | 19.4% | 10 | 4.9% | 35 | 17.2% |
| Grade 2 | 18 | 8.7% | 6 | 2.9% | 13 | 6.4% |
| Grade 3 | 6 | 2.9% | 2 | 1.0% | 5 | 2.5% |
| Grade 4 | 5 | 2.4% | 2 | 1.0% | 3 | 1.5% |
| Missing | 7 | 3.4% | 20 | 9.7% | 10 | 4.9% |

|  | **Outpatient clinic** | | **Phone calls** | | **Blood drawl** | | **ER admission** | | **Hospital admission** | | **Days hospitalized** | | **Transfusions received** | | **Imaging received** | |
| --- | --- | --- | --- | --- | --- | --- | --- | --- | --- | --- | --- | --- | --- | --- | --- | --- |
| Thrombocytopenia | Median | IQR | Median | IQR | Median | IQR | Median | IQR | Median | IQR | Median | IQR | Median | IQR | Median | IQR |
| **CRT - phase** |  |  |  |  |  |  |  |  |  |  |  |  |  |  |  |  |
| Entire population | 0 | (0 – 0) | 0 | (0 – 0) | 0 | (0 – 0) | 0 | (0 – 0) | 0 | (0 – 0) | 0 | (0 – 0) | 0 | (0 – 0) | 0 | (0 – 0) |
| Grade 0 | 0 | (0 – 0) | 0 | (0 – 0) | 0 | (0 – 0) | 0 | (0 – 0) | 0 | (0 – 0) | 0 | (0 – 0) | 0 | (0 – 0) | 0 | (0 – 0) |
| Grades 1 & 2 | 0 | (0 – 0) | 0 | (0 – 0) | 0 | (0 – 0) | 0 | (0 – 0) | 0 | (0 – 0) | 0 | (0 – 0) | 0 | (0 – 0) | 0 | (0 – 0) |
| Grade 3 | 0 | (0 – 0) | 0 | (0 – 1) | 0 | (0 – 1) | 0 | (0 – 0) | 0 | (0 – 1) | 0 | (0 – 1.3) | 0 | (0 – 1.3) | 0 | (0 – 0) |
| Grade 4 | 0 | (0 – 1) | 0 | (0 – 0) | 2 | (0 – 1) | 0 | (0 – 1) | 0.5 | (0 – 1) | 0.5 | (0 – 7) | 1 | (0 – 1.8) | 0 | (0 – 0) |
| **ADJ – phase** |  |  |  |  |  |  |  |  |  |  |  |  |  |  |  |  |
| Entire population | 1 | (0 – 3) | 1 | (0 – 3) | 1 | (0 – 2) | 0 | (0 – 1) | 0 | (0 – 0) | 0 | (0 – 0) | 0 | (0 – 0) | 0 | (0 – 1) |
| Grade 0 | 0 | (0 – 2) | 1 | (0 – 4) | 0 | (0 – 1) | 0 | (0 – 1) | 0 | (0 – 0) | 0 | (0 – 0) | 0 | (0 – 0) | 0 | (0 – 2) |
| Grades 1 & 2 | 1 | (0 – 3) | 1 | (0 – 3) | 1 | (0 – 2) | 0 | (0 – 1) | 0 | (0 – 0) | 0 | (0 – 0) | 0 | (0 – 0) | 0 | (0 – 1) |
| Grade 3 | 2.5 | (1 – 6) | 0.5 | (0 – 4.5) | 4.5 | (3 – 7) | 0 | (0 – 1) | 0 | (0 – 0.3) | 0 | (0 – 0.5) | 0 | (0 – 0) | 0 | (0 – 0.3) |
| Grade 4 | 6 | (3 – 10.5) | 2 | (0 – 5.3) | 10.5 | (8.3 – 13.8) | 0.5 | (0 – 2) | 0.5 | (0 – 1.8) | 1 | (0 – 6.8) | 1 | (1 – 2) | 0 | (0 – 0) |

**Supplementary table 4:**

**Supplementary Table 5:** Results of the generalized linear mixed model examined association between different types of myelotoxicity and unplanned healthcare utilisation during CRT phase.

|  | Exp. Coeff. | t | Sig. | Exp. 95% Confidence Interval | |
| --- | --- | --- | --- | --- | --- |
|  |  |  |  | 2.5% | 97.5% |
| Anemia* |  |  |  |  |  |
| Grade 0 | - | - | - | - | - |
| Grades 1 & 2 | 13.7 | 10.0 | < .001 | 8.2 | 22.9 |
| Grade 3 | 3.1 | 1.9 | .05 | 0.98 | 9.9 |
| Leukocytopenia* | |  |  |  |  |
| Grade 0 | - | - | - | - | - |
| Grades 1 & 2 | 2.6 | 4.3 | < .001 | 1.7 | 3.9 |
| Grade 3 | 1.4 | 1.1 | .28 | 0.74 | 2.8 |
| Grade 4 | 9.0 | 5.7 | < .001 | 4.2 | 19.3 |
| Lymphocytopenia | |  |  |  |  |
| Grade 0 | - | - | - | - | - |
| Grades 1 & 2 | 1.0 | 0.13 | .90 | 0.64 | 1.7 |
| Grade 3 | 4.2 | 1.9 | .057 | 0.96 | 18.7 |
| Grade 4 | 22.6 | 3.5 | .001 | 3.9 | 131.6 |
| Neutropenia |  |  |  |  |  |
| Grade 0 | - | - | - | - | - |
| Grades 1 & 2 | 1.0 | 0.13 | .90 | 0.55 | 2.0 |
| Grade 3 | 44.8 | 7.0 | < .001 | 15.5 | 129.2 |
| Grade 4 | 18.9 | 5.6 | < .001 | 6.7 | 52.8 |

All models are corrected for sex, BSA, use of corticosteroids and tumour side

* Model is additionally corrected for the interaction between myelotoxicity and female sex

**Supplementary Table 6:** Results of the generalized linear mixed model examined association between different types of myelotoxicity and unplanned healthcare utilisation during the adjuvant phase.

|  | Exp. Coeff. | t | Sig. | Exp. 95% Confidence Interval | |
| --- | --- | --- | --- | --- | --- |
|  |  |  |  | 2.5% | 97.5% |
| Anemia |  |  |  |  |  |
| Grade 0 | - | - | - | - | - |
| Grades 1 & 2 | 1.6 | 5.2 | < .001 | 1.3 | 1.9 |
| Grade 3 | 1.3 | 1.5 | .12 | 0.93 | 1.9 |
| Leukocytopenia | |  |  |  |  |
| Grade 0 | - | - | - | - | - |
| Grades 1 & 2 | 0.94 | -0.78 | .44 | 0.82 | 1.1 |
| Grade 3 | 1.0 | 0.26 | .80 | 0.85 | 1.2 |
| Grade 4 | 16.1 | 3.3 | < .001 | 3.1 | 82.1 |
| Lymphocytopenia | |  |  |  |  |
| Grade 0 | - | - | - | - | - |
| Grades 1 & 2 | 3.2 | 3.8 | .000 | 1.8 | 5.7 |
| Grade 3 | 7.0 | 5.4 | < .001 | 3.5 | 14.3 |
| Grade 4 | 3.9 | 2.1 | .03 | 1.1 | 13.9 |
| Neutropenia |  |  |  |  |  |
| Grade 0 | - | - | - | - | - |
| Grades 1 & 2 | 0.81 | -2.2 | .03 | 0.67 | 0.98 |
| Grade 3 | 2.0 | 4.2 | < .001 | 1.5 | 2.8 |
| Grade 4 | 4.5 | 5.6 | < .001 | 2.7 | 7.6 |

All models are corrected for progression of disease. seizure modifying treatment. use of corticosteroids,
use of proton pump inhibitors, tumor side and extend of resection.

**Supplementary Table 7:** Results of the generalized linear model examining the association between the severity of thrombocytopenia and ER admissions and days spent hospitalized.

|  | Exp. Coeff. | t | Sig. | Exp. 95% Confidence Interval | |
| --- | --- | --- | --- | --- | --- |
|  |  |  |  | 2.5% | 97.5% |
| Days spent admitted during CRT phase* |  |  |  |  |  |
| *Thrombocytopenia* |  |  |  |  |  |
| Grade 0 | - | - | - | - | - |
| Grade 1 & 2 | 3.0 | 2.8 | .005 | 1.4 | 6.3 |
| Grade 3 | 3.3 | 5.3 | < .001 | 3.5 | 14.8 |
| Grade 4 | 2.5 | 1.3 | .21 | 0.6 | 10.8 |
| *Interaction female sex by grade thrombocytopenia* |  |  |  |  |  |
| Grade 0 | - | - | - | - | - |
| Grade 1 & 2 | 1.0 | 0.03 | .97 | 0.15 | 7.2 |
| Grade 3 | 7.2 | 5.3 | < .001 | 3.5 | 14.8 |
| Grade 4 | 21.3 | 3.0 | .003 | 2.8 | 162.7 |
| Days spent admitted during ADJ phase** |  |  |  |  |  |
| *Thrombocytopenia* |  |  |  |  |  |
| Grade 0 | - | - | - | - | - |
| Grade 1 & 2 | 5.5 | 5.7 | < .001 | 3.1 | 9.9 |
| Grade 3 | 0.3 | -1.6 | .11 | 0.06 | 1.3 |
| Grade 4 | 3.1 | 3.4 | .001 | 1.6 | 6.1 |
| *Interaction female sex by grade thrombocytopenia* |  |  |  |  |  |
| Grade 0 | - | - | - | - | - |
| Grade 1 & 2 | 0.19 | -4.3 | < .001 | 0.09 | 0.41 |
| Grade 3 | 5.4 | 5.1 | < .001 | 2.8 | 10.4 |
| Grade 4 | 1.1 | -0.21 | .83 | 0.43 | 2.0 |
| ER presentations during CRT phase*** |  |  |  |  |  |
| *Thrombocytopenia* |  |  |  |  |  |
| Grade 0 | - | - | - | - | - |
| Grade 1 & 2 | 1.4 | 0.84 | .40 | 0.63 | 3.1 |
| Grade 3 | 3.1 | 1.8 | .07 | 0.93 | 10.2 |
| Grade 4 | 4.3 | 3.7 | < .001 | 1.98 | 9.2 |
| ER presentations during ADJ phase**** |  |  |  |  |  |
| Grade 0 | - | - | - | - | - |
| Grade 1 & 2 | 0.96 | -0.11 | .92 | 0.93 | 1.9 |
| Grade 3 | 0.91 | 0.13 | .90 | 0.21 | 3.8 |
| Grade 4 | 2.27 | 1.8 | .08 | 1.09 | 5.6 |

* Model corrected for female sex, interaction between sex and thrombocytopenia and tumour side

** Model corrected for female sex, interaction between sex and thrombocytopenia and progression of disease

*** Model not corrected for other variables

**** Model corrected for progression of disease
